# Phase angle as an indicator of body composition and physical performance in handball players

**DOI:** 10.1186/s13102-024-00899-1

**Published:** 2024-05-21

**Authors:** Valmir Oliveira Silvino, Kelly Raffaela Barbosa Barros, Felipe Machado Brito, Francisco Matheus Dias Magalhães, Antônio Augusto Ferreira Carioca, Adriano César Carneiro Loureiro, Acácio Salvador Veras-Silva, Marcos Daniel Motta Drummond, Marcos Antonio Pereira dos Santos

**Affiliations:** 1 Department of Biophysics and Physiology, Federal University of Piaui, 3700 Pedro Freitas Avenue, Teresina, 64018000 Piauí Brazil; 2 Nucleus of Study in Physiology Applied to Performance and Health (NEFADS), Federal University of Piaui, Teresina, Piauí Brazil; 3 Rede Nordeste de Biotecnologia (RENORBIO), Federal University of Piauí, Teresina, Piauí Brazil; 4 Nutrition Course , Health Sciences Center, University of Fortaleza, Fortaleza, Ceará Brazil; 5 Department of Physical Education, State University of Ceará, Fortaleza, Ceará Brazil; 6 Department of Physical Education, Physiotherapy and Occupational Therapy, Federal University of Minas Gerais, Belo Horizonte, Minas Gerais Brazil

**Keywords:** Bioimpedance analysis, Body composition, Vertical jump, Handgrip strength, Aerobic performance

## Abstract

**Background:**

Phase angle (PhA), obtained from the bioimpedance analysis, is widely used in clinical situations and in sports. This study evaluated the association between PhA with body composition and physical performance of handball athletes.

**Methods:**

43 national-level players (22.19 ± 3.86 years) of both sexes were evaluated regarding anthropometry, body composition, squat (SJ) and countermovement (CMJ) jumps, handgrip strength, and cardiorespiratory fitness.

**Results:**

We verified a correlation between PhA of the whole body and fat-free mass (*r* = 0.511), body mass index (*r* = 0.307), and body fat % (*r* = -0.303). There was a positive correlation between PhA of the whole body and SJ (*r* = 0.376), CMJ (*r* = 0.419), and handgrip for the dominant hand (*r* = 0.448). Moreover, PhA of the upper limbs was more strongly correlated with handgrip for the dominant (*r* = 0.630) and non-dominant hand (*r* = 0.575) compared to PhA of the whole body considering both sexes. Similarly, segmental PhA had a stronger significant correlation with SJ (*r* = 0.402) and handgrip for the dominant hand (*r* = 0.482) in males, as well as CMJ (*r* = 0.602) in females, compared to PhA of the whole body.

**Conclusion:**

PhA of the whole body was positively related to fat-free mass, body mass index, body fat %, and lower- and upper-limbs strength in handball athletes. Segmental PhA might be used as a tool for estimating lower and upper limbs performance considering the sex, in preference to the PhA of the whole body.

## Background

Handball is a sport that requires high-intensity and short-duration physical efforts with vigorous contact and repeated explosive muscle contractions [1]. It is characterized by repeated jumps, sprints, changes in direction, physical contact at high speed, and specific technical movement patterns [2], interspersed with actions necessary for recovery, such as walking and standing [3]. Thus, handball requires specific training programs to improve conditioning, including high-intensity exercises such as resistance training to enhance these physical qualities [4]. Due to the complex nature of the sport, the physical demands in handball vary depending on factors such as playing position, the level of competition, and gender. These demands encompass various physical aspects, including the strength of both upper and lower limbs, as well as cardiorespiratory fitness [5].

The analysis and monitoring of body composition is essential in sports [6, 7], since body composition is directly related to the increase in aerobic and anaerobic performance and muscle strength in handball athletes [8]. Among the procedures used to assess body composition, electric bioimpedance analysis (BIA) stands out [9], as it is a safe, fast, and non-invasive method to estimate body composition in different populations. Several BIA devices assess body resistance and reactance by analyzing electrical impedance across different tissues. The process involves applying electrical currents to the body, which initially disperse through areas with high water content, including both intracellular and extracellular water. As the current frequency increases, it traverses through body water content due to changes in the capacitive effect of cell membranes. By applying Ohm’s law, the voltage difference recorded between hands divided by the current intensity produces the resistance of the arm to the electrical currents [10]. This technique enables the measurement of segmental body composition through built-in predictive equations, evaluating several parameters, such as fat-free mass (FFM), body cell mass, total body water, extracellular water, and intracellular water [6, 11]. The most innovative utilization of BIA involves assessing the basic bioelectrical parameters via vector analysis known as bioelectrical impedance vector analysis (BIVA). Essentially, bioimpedance is seen as the combined outcome of bioelectrical resistance (R) and reactance (Xc) [12].

The electrical current passing through cellular membranes, functioning as capacitors, causes a phase shift referred to as the geometric phase angle (PhA) [13]. The calculation of the PhA is performed from the primary values of R and Xc of the bioimpedance. This parameter expresses the electrical function of cell membranes and reflects the proportion between extracellular and intracellular water in body compartments [6, 14, 15]. Factors such as age, gender, and body mass index (BMI) significantly influence PhA in healthy adults. With advancing age, PhA typically decreases as resistance rises due to a decline in body water ratio and an increase in fat mass. Men tend to exhibit a higher PhA compared to women, primarily due to greater muscle mass [16]. Thus, PhA has been widely used as an indicator of cell health, cell membrane integrity, and cell function, not only in the general population but also in athletes [9, 11, 17, 18].

It is well known in the literature that physical exercise directly influences the PhA of athletes. This is due to the fact that physical training reflects in greater FFM values, leading to the increase in intracellular water, which reduces resistance and, consequently, increases in PhA [11, 14, 19]. The complementary use of BIA and PhA in sports contexts may be useful for the assessment of changes in body composition and performance evaluation. We hypothesize that PhA can be used by coaches and athletes as a tool to estimate body composition and performance status in handball players. Therefore, the objective of this study is to evaluate the relationship between phase angle with body composition and sports performance in handball athletes of national level.

## Methods

### Participants

The study was carried out with 43 national-level handball athletes (28 men and 15 women) aged over 18 years (22.19 ± 3.86 years). They had been training for at least 1 year with a weekly frequency of five training sessions, 90–120 min per day. Additionally, the participants participated regularly in international and national competitions in the previous 2 years. The exclusion criteria were: having any chronic degenerative disease, endocrine and/or thermoregulatory disorders, smokers, alcoholics, users of any medication or vitamin-mineral supplements that would alter the hydroelectrolyte balance, dysfunctions in the health history or other problem that could compromise the physical integrity of the volunteers. Participants who were ingesting any substance which could enhance their performance or body composition were removed from the study. A post hoc sample power of 0.52 was calculated using the GPower software, considering the following specifications: α = 0.05, effect size = 0.3 and total sample size = 43. All participants signed the Informed Consent Term, in accordance with Resolution 466/12 of the National Health Council. The research was approved by the Research Ethics Committee protocol number 5.134.334.

### Anthropometric measurements

Body mass was measured using a Filizola ® digital scale (São Paulo, Brazil), with a capacity of 150 kg, graduated in 100 g. Stature was measured at anatomical position in duplicate using a portable stadiometer (Sanny Standard, São Paulo, Brazil) with a measurement scale of 0.1 cm. The participants wore light clothing and no shoes during the measurements. Body mass index (BMI) was calculated through the coefficient Body mass (kg) / stature (m^2^).

### Bioimpedance analysis

Body composition parameters (body fat %, FFM and PhA) were assessed using a multi-tactile impedancemeter with eight electrodes at a frequency of 50 kHz (InBody S10, Biospace, Seoul, Korea). InBody S10 is a validated method for estimating skeletal muscle mass in young subjects in comparison to dual-energy X-ray absorptiometry, which is considered the gold standard measure [20]. Bioimpedance analysis was conducted with the volunteers at supine position. The participants were asked to refrain from exercise 12 h prior, diuretics or caffeine 12 h prior, and food or liquids intake 30 min prior to the measurements. Participants in their menstrual period would have to notify the researchers and reschedule the exam 24 h before the measurements. They had their skin cleaned with alcohol and the touch type electrodes were connected to the thumb and the middle fingers of the right and left arm and between the anklebone and heel of the right and left leg. The participants were instructed to maintain a supine position for approximately 10 to 15 min before the test to ensure even distribution of body water throughout the body. We ensured that their arms were positioned away from the trunk, naturally spreading them to a 15-degree angle. Additionally, we verified that their thighs were not in contact with each other, spreading their legs to shoulder width apart. The measurements were conducted in the morning, within a room environment where the ambient temperature and relative humidity were maintained at 22–23 °C and 50–60%, respectively. Furthermore, FFM was estimated using total body water content, from which fat mass was subsequently calculated [21]. Additionally, PhA, determined as the ratio of electric reactance to electric resistance, was computed using the following Eq. [22]:

$$PhA=arctangent \left(\frac{Xc}{R}\right)\times 180^\circ /\pi$$

### Lower limbs strength

Vertical jump analysis, commonly used for assessing athletic performance [23], was carried out to estimate the strength of the lower limbs. Participants performed 3 squat jumps (SJ) (hands on waist and knee flexion at 90° in the starting position) and 3 countermovement vertical jumps (CMJ) (hands on waist and use of knee flexion countermovement in position for the take-off at the start of the jump). The jump height was estimated using the previous validated [24] My Jump 2 application (Apple Inc., USA). For evaluation, the length of the lower limbs was measured in the upright position (distance between the greater trochanter of the femur and the tip of the foot in plantar flexion), then with the knees flexed at approximately 90°. The interval between jumps was 1 min. The jumps were filmed at a distance of approximately two meters from the participants.

### Aerobic performance

The 30 − 15 test was conducted to estimate maximal oxygen consumption (VO_2_max), determine maximum heart rate and anaerobic and intermittent capacity [2], widely used in several sports, including handball, futsal, basketball and soccer [25]. The test consists of 30-second runs interspersed with 15-second passive recovery periods. The test application followed the protocol used by Buchheit [25]. VO_2_max was estimated using the values of sex (S; female = 2; male = 1), age (A), body mass (BM), and the final speed reached at the end of the 30–15 test (FST), using the following equation:

$$\begin{gathered} V{O_2}max = 28.3 - 2.15 \times S - 0.741 \times A \\ - 0.0357 \times BM + 0.058 \times I \times FST + {\text{1,02}} \times FST \\ \end{gathered}$$

### Hand grip strength

To assess muscle strength of the upper body, handgrip strength was verified using a handheld dynamometer. The measurement was performed by a trained evaluator using the Crown® 100 kgf / 1 kgf dynamometer. Patients performed the test at rest in a horizontal position, with arms extended by their sides and forearms and wrists in a neutral position. Subjects were instructed to perform three maximal isometric contractions, with a brief pause (30 s) between measurements. Three measurements were taken for each hand (dominant and non-dominant). The handgrip strength measurement values were grouped into dominant hands and non-dominant hands. The mean of the three measurements was used, shown in kilograms/force [26].

### Statistical analysis

Data normality was assessed using the Shapiro-Wilk test. Student t test was used to compare means between male and female participants. Pearson’s correlation was used to analyze the correlation between independent variables and dependent variables. The magnitude of correlation between tests was evaluated using the following thresholds: < 0.3 negligible; >0.3 to 0.5 weak; >0.5 to 0.7 moderate; >0.7 to 0.9 strong; >0.9 very strong; 1 perfect [27]. For all statistical analyses, significance was accepted at *p* < 0.05. Data were analyzed using SPSS software version 20.0 (SPSS, Inc., Chicago, IL, USA).

## Results

Table 1 shows the characteristics of the participants with body composition data (body mass, BMI, body fat percentage, fat mass, FFM, and PhA) and performance parameters (vertical jumps, VO_2_max, and handgrip tests).

**Table 1:** Anthropometric characteristics of the participants

Variables	Male (*N* = 28)	Female (*N* = 15)	*p*	All participants (*N* = 43)
**Body mass (kg)**	79.86 ± 14.06	63.65 ± 8.23	0.001*	74.2 ± 14.5
**Age (years)**	23.21 ± 4.05	20.26 ± 2.63	0.015*	22.8 ± 3.8
**BMI (kg/m²)**	24.68 ± 3.68	23.90 ± 3.38	0.497	2.4 ± 3.6
**Body fat (%)**	16.19 ± 6.93	26.45 ± 7.25	0.001*	19.8 ± 8.5
**Fat mass (kg)**	13.52 ± 7.99	17.27 ± 6.83	0.132	14.8 ± 7.7
**Fat-free mass (kg)**	64.83 ± 10.49	41.68 ± 2.96	0.001*	56.8 ± 14.1
**Phase angle (º)**	7.11 ± 0.53	6.51 ± 0.49	0.001*	6.9 ± 0.6
**Squat jump (cm)**	35.61 ± 7.38	24.63 ± 4.62	0.001*	31.7 ± 8.4
**Countermovement jump (cm)**	37.21 ± 7.04	24.08 ± 5.56	0.001*	32.6 ± 9.1
**Handgrip dominant hand (kg)**	46.75 ± 6.19	27.05 ± 5.04	0.001*	39.9 ± 11.1
**Handgrip non-dominant hand (kg)**	42.07 ± 9.93	26.41 ± 7.32	0.001*	36.4 ± 11.9
**VO**_**2**_ **max (ml/kg/min)**	47.61 ± 5.11	40.07 ± 3.89	0.001*	45.7 ± 5.8
**30 − 15 speed (km/h)**	17.39 ± 2.06	15.06 ± 1.61	0.007*	16.8 ± 2.2

*, statistical difference (*p* < 0.05). Legends: BMI, body mass index; VO_2_max, maximal oxygen consumption; SD, standard deviation of the mean

**Fig. 1:**
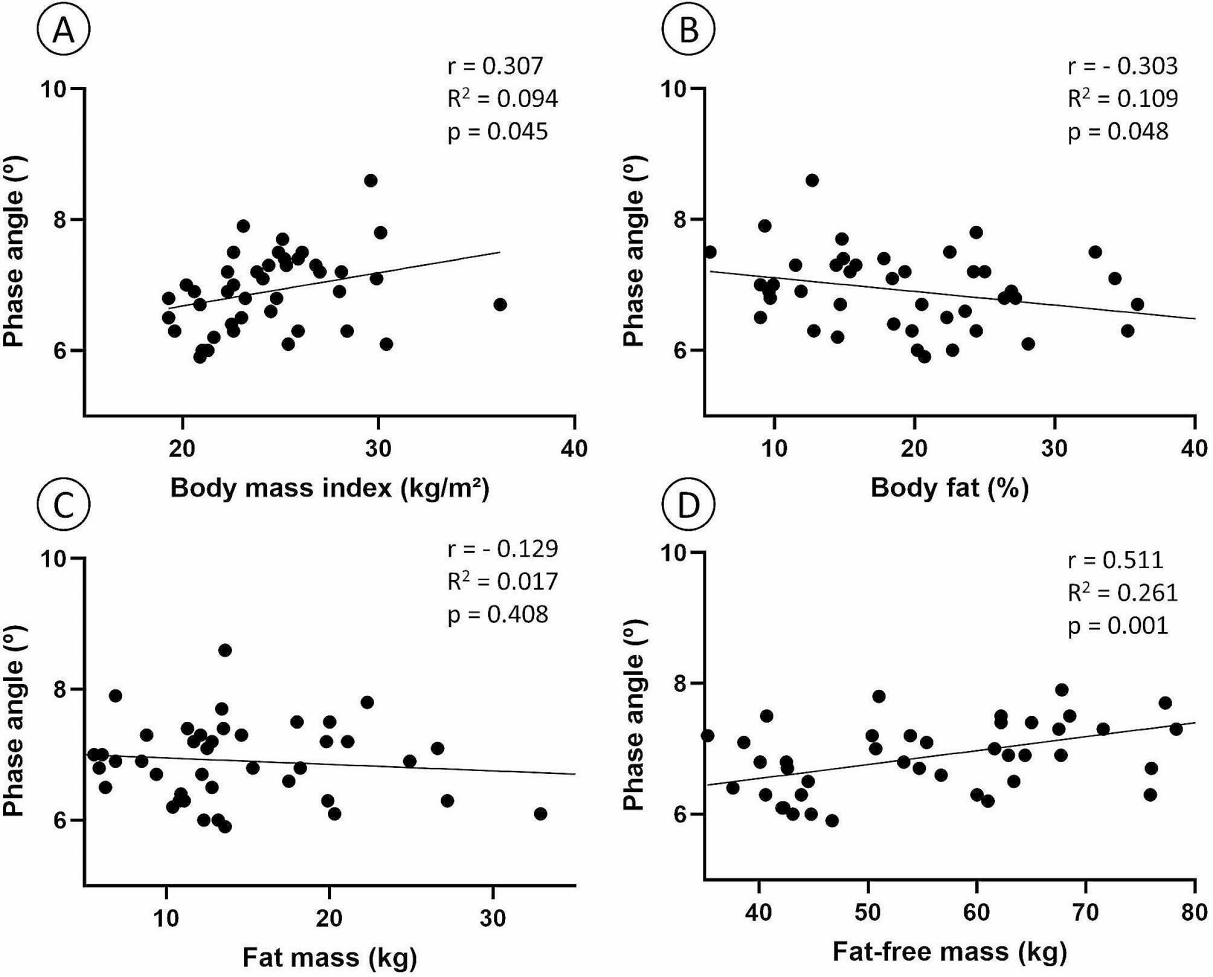
Correlation between phase angle of the whole body and body mass index (Panel **A**), body fat percentage (Panel **B**), fat mass (Panel **C**), and fat-free mass (Panel **D**) considering all participants

The correlation between PhA and body composition and physical performance variables are presented in Table 2 considering each sex. Concomitantly, Fig. 1 shows a positive and significant correlation between PhA and BMI, body fat %, and FFM considering all participants (*p* < 0.048).

**Table 2:** Correlation between phase angle of the whole body and body composition and physical performance parameters

Phase angle	Male		Female	
	*r* value	*p* value	*r* value	*p* value
**BMI**	0.340	0.076	0.190	0.498
**Body fat %**	-0.135	0.492	0.198	0.478
**Fat mass**	-0.050	0.800	0.066	0.816
**Fat-free mass**	0.361	0.059	0.634	0.011*
**Squat jump**	0.271	0.163	0.499	0.058
**Countermovement jump**	0.342	0.075	0.472	0.076
**Handgrip dominant hand**	0.212	0.280	0.341	0.213
**Handgrip non-dominant hand**	-0.118	0.559	0.074	0.795
**30 − 15 speed**	-0.002	0.991	0.437	0.279
**VO**_**2**_ **max**	-0.106	0.621	0.461	0.250

*Significant correlation, *p* < 0.05. Legends: BMI, body mass index; PhA, Phase angle; VO_2_max, maximal oxygen consumption

**Fig. 2:**
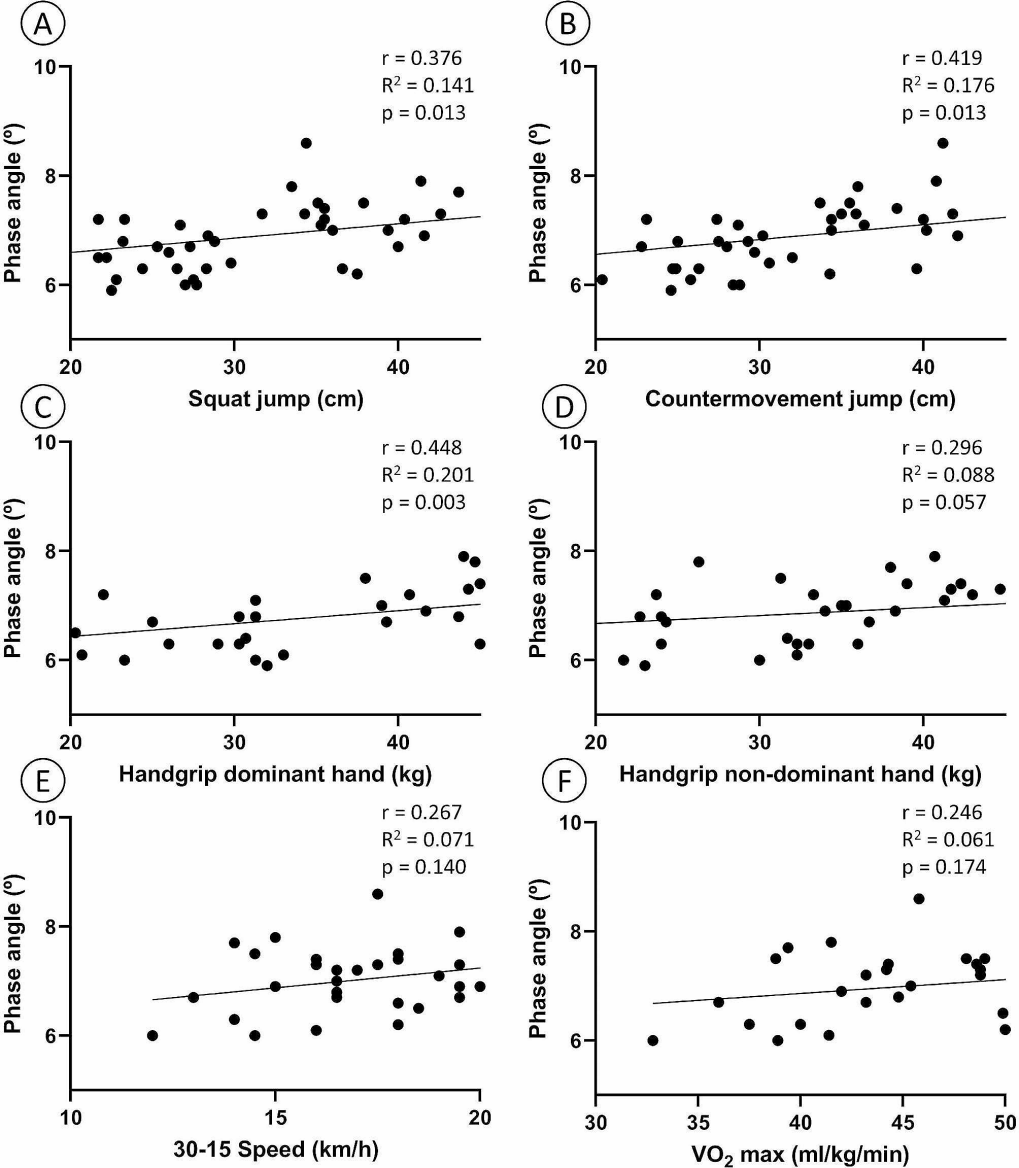
Correlation between phase angle of the whole body and squat jump (Panel **A**), countermovement jump (Panel **B**), handgrip strength of the dominant hand (Panel **C**), handgrip strength of the non-dominant hand (Panel **D**), final speed at the end of the 30 − 15 test (Panel **E**), and maximal oxygen consumption (Panel **F**) considering all participants

A statistically significant and positive correlation was identified between the PhA and SJ, CMJ, and handgrip strength for the dominant hand considering all participants (*p* < 0.013), as seen in Fig. 2.

Table 3 shows the correlation between PhA of the upper and lower limbs and the muscle strength of upper and lower limbs, respectively, for each sex and considering all participants.

**Table 3:** Correlation between phase angle of lower and upper limbs and the physical performance

Sex	Phase angle	Variables	*r* value	*p* value
Overall	PhA upper limbs	Handgrip dominant hand	0.630	0.001*
		Handgrip non-dominant hand	0.575	0.001*
	PhA lower limbs	Squat jump	0.300	0.049*
		Countermovement jump	0.234	0.131
Males	PhA upper limbs	Handgrip dominant hand	0.428	0.026*
		Handgrip non-dominant hand	0.121	0.548
	PhA lower limbs	Squat jump	0.402	0.034*
		Countermovement jump	0.350	0.068
Females	PhA upper limbs	Handgrip dominant hand	-0.075	0.790
		Handgrip non-dominant hand	0.320	0.245
	PhA lower limbs	Squat jump	0.506	0.607
		Countermovement jump	0.602	0.016*

*Significant correlation, *p* < 0.05. Legends: PhA, Phase angle

## Discussion

The present study aimed to depict the association between the phase angle and body composition status and physical performance variables in handball athletes of national level. The hypothesis that PhA can be a predictor of optimal body composition and physical performance in handball athletes was confirmed. The main findings of this study were that greater PhA levels was significantly correlated with FFM, body mass index, body fat %, vertical jump height and handgrip strength when considering all subjects. Secondarily, we found that the segmental PhA of the upper and lower limbs had a stronger relationship with upper and lower limbs strength, respectively, when compared with the PhA of the whole body for both sexes. Likewise, segmental PhA was more strongly correlate with SJ and handgrip for the dominant hand performance than PhA of the whole body in male athletes, as well as CMJ in females.

We observed a positive and statistically significant relationship between PhA and FFM considering the analysis with all participants. This corroborates with the current literature, which states that FFM, an indicator of physical fitness and nutritional status in athletes of different sport disciplines, is the strongest predictor of PhA, even stronger than BMI or body fat [7, 28, 29]. Indeed, studies have indicated that lower PhA values are associated with higher levels of body fat, indicating a potential link between reduced cellular health and increased adiposity [30, 31]. Likewise, we observed a negative correlation between body fat % and PhA when evaluating both sexes. However, it is essential to recognize that PhA is influenced by various factors beyond body fat alone, such as hydration status, muscle mass, and overall health conditions [32]. Therefore, while phase angle may offer insights into body composition and health status, it should be interpreted alongside other clinical measures to provide a comprehensive assessment.

In the present study, a significant positive correlation was identified between PhA and SJ, CMJ and handgrip for the dominant hand when assessing all participants. It is worth mentioning that, when examining each sex individually, PhA of the whole body was significantly correlated only with FFM in females. Indeed, physical training can lead to an increase in intracellular water, especially when it causes an increase in muscle mass. This is due to the fact that physical training reduces resistance and, consequently, leads to an increase in PhA, thus explaining its correlation with FFM [33–35]. Health-related physical fitness is usually assessed through musculoskeletal fitness (strength, power, and endurance) and is analyzed through tests such as hand grip strength [36] and vertical jump tests [37]. Higher PhA can be found in individuals with higher muscle mass, and although the direct association between muscle mass and strength is currently debated, muscle mass is still one of the main contributing factors to strength [29, 34, 38].

One of the findings of this study was that the segmental PhA were more strongly correlated with upper and lower limbs performance when compared with the PhA of the whole body. This indicates that segmental PhA could serve as a useful indicator for predicting muscle strength in handball athletes, rather than relying on the PhA of the entire body. Similarly, Bongiovanni et al. [39] concluded that CMJ performance is more strongly related to the PhA of the lower limbs than the PhA of the whole body in elite soccer players. However, Hetherington-Rauth et al. [40] evaluated segmental and whole body PhA in 117 adults from different sports and concluded that the segmental PhA of upper and lower limbs were not a better indicator of strength and power than the PhA of the whole body.

We observed in our study that PhA was not a predictor of VO_2_max. One conceivable explanation involves cellular mass and FFM at the molecular level, as both factors have been noted to correlate directly with PhA [6, 41]. A systematic review concluded that PhA is directly associated with muscle strength and aerobic fitness in different age groups and in people with different health conditions [42]. It has been also verified a positive correlation between PhA and anaerobic performance parameters in soccer players [41], as well as aerobic fitness in futsal players [43].

PhA has been shown to have practical applications in assessing physical performance in handball athletes. Thus, it can be used by athletes and trainers in order to estimate athletic status. However, the present study has limitations that must be acknowledged. This study used a cross-sectional design that does not allow the establishment of a cause-effect relationship. Further studies with different sports and longer period of food intake report should be carried out.

## Conclusion

The findings of this study found that PhA is associated with fat-free mass, body mass index, body fat % and upper and lower muscle strength in national-level handball players. Furthermore, we found that, considering the sex, segmental PhA was a better predictor of upper and lower body muscle strength when compared to the PhA of the whole body. Therefore, segmental PhA could be used to predict muscle strength performance in handball athletes in preference to the PhA of the whole body.

## Data Availability

The datasets generated and/or analysed during the current study are available from the corresponding author on reasonable request.

## Abbreviations

BIA: Bioimpedance analysis
BM: Body mass
BMI: Body mass index
CMJ: Countermovement jump
PhA: Phase angle
SD: Standard deviation
SJ: Squat jump
VO_2_ max: Maximal oxygen consumption
